# The Application of Tortoise-Shell to Mechanical Dentistry

**Published:** 1850-07

**Authors:** James Robinson


From Am. Jour. of Dental Science.

### THE APPLICATION OF TORTOISE-SHELL TO MECHANICAL DENTISTRY.

BY DR. JAMES ROBINSON.

A LARGE number of the dental profession attended, by invitation, at the Western Literary Instiution, Leicester Square, London, on Tuesday, the 5th February, 1850, to hear a lecture by Mr. G. F. Harrington, Dentist of Portsmouth, on the application of tortoise-shell for the beds of artificial teeth, the following is the substance of the lecturer's observations.

The lecturer and patentee of this new application introduced the subject, by observing, that as the gentlemen composing the audience were well acquainted with the theory and practice of the dental art, he would not occupy their time with any preliminary remarks, but at once proceed to describe the nature and peculiar advantages of tortoise-shell over that of sea-horse or sea-cow ivory, as the basis for artificial teeth, both as regards cleanliness, strength and durability, it was, moreover, only half the weight of the former materials, and when seen in connection with the mineral teeth, its advantages would be obvious.

As regarded cleanliness, it stood unrivalled, with the exception of gold, as it possessed neither taste nor smell, nor was there any perceptible change from the action of the saliva of the mouth, neither was there any chemical change produced when submitted to the action of various acids with which he had experimented. It, moreover, was a nonconductor of heat and cold, and hence, excellently adapted for tender gums.

The working of the material was exceedingly easy, as it could be readily filed, scraped, drilled and riveted without the least danger of accident; it was non-absorbent and capable of receiving and retaining a high polish, and from the results of Mr. H.'s experiments for five years, he assured the meeting that it would be found more durable than either of the ivorys usually employed, and probably equal to gold in that respect.*

At present the application is limited to full sets in either jaw, whether its extension to other cases remains to be seen. The method of taking the impression is very ingenious and interesting. It consists of a series of hollow model sets manufactured in metal and numbered, which externally represent teeth of the various width and depth usually required; this tray is filled with wax and pressed upon the gum to the proper depth, in the usual manner. The patient is now desired to close the mouth, which gives the requisite length, &c. By this means the lecturer stated the model, bite and adaptation in complete sets could be finished at one interview with the patient.

The operator has now to submit his model to a graduated guage, invented for the purpose of taking the depth and width of his artificial substitutes, and as before observed, a number of sizes and shapes are kept ready manufactured by Messrs. T. Ash & Co., corresponding to the number of his tray, by means of which he took the impression in the first instance. After selecting the one adapted to the case, it is mounted upon the tortoise-shell palate plates by means of rivets, the porcelain gum attached to the teeth, in most instances, extending sufficiently high up to prevent the color of the shell being observable externally. The operator has now merely to attach his swivel and springs, and the set is ready for insertion in the patient's mouth.

Throughout the delivery of the lecture, Mr. H. was listened

---

* Not having the drawings of the machines for compressing, the descriptions which accompany are also omitted.—Ed.

to with great attention, and at its conclusion, offered to explain any part of his process to any person interested, which we understand many availed themselves of.

